# On the Relationship between Value- and Threat-Driven Attentional Capture and Approach-Avoidance Biases

**DOI:** 10.3390/brainsci13020158

**Published:** 2023-01-17

**Authors:** Haena Kim, Brian A. Anderson

**Affiliations:** Department of Psychological and Brain Sciences, Texas A&M University, 4235 TAMU, College Station, TX 77843-4235, USA

**Keywords:** selection history, selective attention, reward learning, aversive conditioning, approach, avoidance, action

## Abstract

Reward learning and aversive conditioning have consequences for attentional selection, such that stimuli that come to signal reward and threat bias attention regardless of their valence. Appetitive and aversive stimuli have distinctive influences on response selection, such that they activate an approach and an avoidance response, respectively. However, whether the involuntary influence of reward- and threat-history-laden stimuli extends to the manner in which a response is directed remains unclear. Using a feedback-joystick task and a manikin task, which are common paradigms for examining valence-action bias, we demonstrate that reward- and threat-signalling stimuli do not modulate response selection. Stimuli that came to signal reward and threat via training biased attention and invigorated action in general, but they did not facilitate an approach and avoidance response, respectively. We conclude that attention can be biased towards a stimulus as a function of its prior association with reward or aversive outcomes without necessarily influencing approach vs. avoidance tendencies, such that the mechanisms underlying the involuntary control of attention and behaviour evoked by valent stimuli can be decoupled.

## 1. Introduction

Reward learning and aversive conditioning have consequences for attentional selection [1]. Stimuli repeatedly paired with reward and aversive outcomes acquire the ability to capture attention, even when they are task-irrelevant and physically non-salient (referred to as value- and threat-driven attentional capture; e.g., [2,3]). Both reward and threat histories develop via associative learning [4,5,6,7], and they also share a common neural profile [8]. Such similarities between the two influences are considered evidence for the motivational salience account, which claims that the attentional system is primarily guided by relevance-for-survival, rather than a particular emotional valence [9,10,11].

Automatic attentional orienting facilitates not only the detection of reward- and threat-related stimuli, both of which have relevance-for-survival, but also the preparation of a subsequent action [12,13]. Indeed, reward learning and aversive conditioning also have consequences for action [14]. Stimuli that come to signal either a reward or an aversive outcome via training are known to influence inhibitory control even when presented as task-irrelevant distractors, either facilitating [15,16] or impairing response suppression [17,18,19]. However, how the involuntary influence of the reward and threat histories might extend to the manner in which a response is directed remains unclear.

Research on valence-action biases suggests that reward and threat histories may guide behaviour in divergent directions. After the detection of motivationally salient stimuli, in the action preparation/execution stage, appetitive and aversive stimuli exert distinct influences, such that the appetitive and aversive stimuli evoke approach and avoidance actions, respectively [20,21,22]. Importantly, such modulation of the response direction by valence is inherent in nature [13,23]. These findings propose that stimuli that come to signal outcomes of opposing valence would also automatically modulate the response direction, with reward-signalling stimuli activating an approach action and threat-signalling stimuli activating an avoidance action. In fact, it has been demonstrated that a selection history shaped via repeated exposure influences the response direction [24]. However, given the distinct nature underlying a selection history shaped via repetition in contrast to reward and threat histories [1,5,25], the relationship between value- and threat-driven attentional capture and biases in the response direction remains to be clarified.

A key brain region Implicated in the detection-preparation/execution account is the basal ganglia. In the detection phase, the caudate tail responds preferentially to stimuli signalling reward and aversive outcomes [8,26], and an increased dopamine level in the caudate predicts a stronger value-driven attentional capture [27,28]. Such reward and threat histories develop via plasticity within the caudate tail, which is guided by dopaminergic projections from the caudal-dorsolateral substantia nigra pars compacta [29,30]. Once learning is complete, both reward- and threat-history-laden stimuli trigger attentional orienting by modulating inhibitory projections in the caudate tail-substantia nigra pars reticulata-superior colliculus circuit [31,32]. The basal ganglia also play a role in motor control via direct and indirect pathways [33], which share similar characteristics with the attentional orienting circuit. The direct pathway guides approach action and the indirect pathway guides avoidance action, in response to appetitive and aversive stimuli, respectively [34,35]. In addition, motor learning is believed to involve a similar value-based plasticity within the caudate tail [36]. Such a shared neural profile between attentional orienting and motor control suggests that reward and threat histories also have a potential to bias the response direction, even to stimuli that are currently task-irrelevant.

Additional support for the hypothesis that reward and threat histories would also modulate the response direction can be found in the amygdala. Although it was traditionally believed to specialise in the processing of negative emotions [37,38], there is growing evidence of its role in appetitive stimulus processing [39]. In the detection phase, the amygdala facilitates attentional orienting to not only aversive but also to appetitive stimuli [40,41,42,43] by modulating inhibitory projections in the circuit for attentional orienting [44], consistent with the motivational salience account. In the preparation/execution phase, different populations of neurons in the basolateral amygdala modulate the response direction via their projections to the central amygdala, which in turn acts on the periaqueductal grey such that approach action is facilitated in response to appetitive stimuli and avoidance action is facilitated in response to aversive stimuli [45,46,47,48].

In summary, based on the prior findings that (1) detection of motivationally salient stimuli facilitates the preparation of an appropriate action, (2) the approach-avoidance tendency is hard-wired, and (3) there is an overlap between the neural mechanisms of reward- and threat-driven attention and action preparation/execution, we hypothesised that reward and threat histories would exert distinct effects on the response direction: reward-signalling stimuli would generate an approach action, whereas threat-signalling stimuli would generate an avoidance action. In particular, we focus on the influence of reward- and threat-signalling distractors on the control of the response direction. Modulation of the response direction by a task-irrelevant distractor would be considered a genuine indication of the involuntary nature of the reward- and threat-driven action.

## 2. Experiment 1

Experiment 1 closely followed Chen and Bargh’s [20] task design to shape reward and threat histories. Participants were instructed to pull a joystick to approach and push it to withdraw in response to a target circle. As participants either pushed or pulled, the target circle became either larger or smaller, producing a perceptual effect that it was pulled closer or pushed away [49]. If reward and threat histories involuntarily modulate the response direction, reward-signalling stimuli should activate an approach action and threat-signalling stimuli should activate a withdrawal action. Participants first completed a training phase in which colour-outcome relationships could be learned from experience in the task, which also provided an opportunity to examine the modulatory effect of these relationships on approach and avoidance behaviour (when the target required a pull and push response, respectively). A subsequent test phase, in which rewards and shocks were no longer delivered and the colour of the stimuli was task-irrelevant, provided an opportunity to address our primary research question of whether reward- and threat-signalling stimuli involuntarily activate approach and avoidance response biases. We were particularly interested whether a previously reward- and shock-associated distractor differently facilitated the approach and avoidance responses required of the target.

### 2.1. Methods

#### 2.1.1. Participants

Thirty-nine participants (19 females; mean age = 22.3 years) were recruited from the Texas A&M University community. All participants had normal or corrected-to-normal visual acuity and normal colour vision, and all were right-handed. All procedures were approved by the Texas A&M University Institutional Review Board and conformed with the principles outlined in the Declaration of Helsinki.

#### 2.1.2. Apparatus

A standard Windows computer equipped with MATLAB software and Psychophysics Toolbox [50] was used to present the stimuli. The eye-to-screen distance was approximately 70 cm. Responses were entered using a Logitech G Extreme 3D Pro Joystick. Electric shocks were generated by an isolated linear stimulator (BIOPAC) operating in current mode.

#### 2.1.3. Procedure

##### Shock Calibration

Electric shocks were delivered via electrodes attached to participants’ left forearm. The shock intensity was adjusted by gradually increasing it to a level where participants perceived it as uncomfortable but not painful (as in, e.g., [3,6]).

##### Training Phase

**Figure 1:**
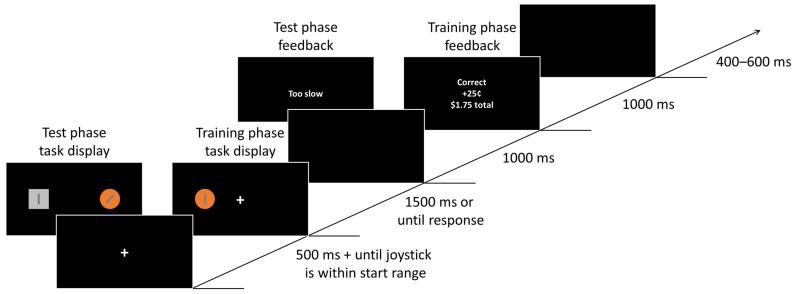
In Experiment 1, the sequence of trial events was identical for the training and test phases, except that, during the training phase, the task display only contained a target circle, and feedback was always provided (“Correct” or “Incorrect”), together with outcome delivery. During the test phase, the task display contained a target square and a distractor circle, one on each side. Feedback was provided only when participants made an incorrect response (“Incorrect”) or failed to make a response in time (“Too slow”).

**Figure 2:**
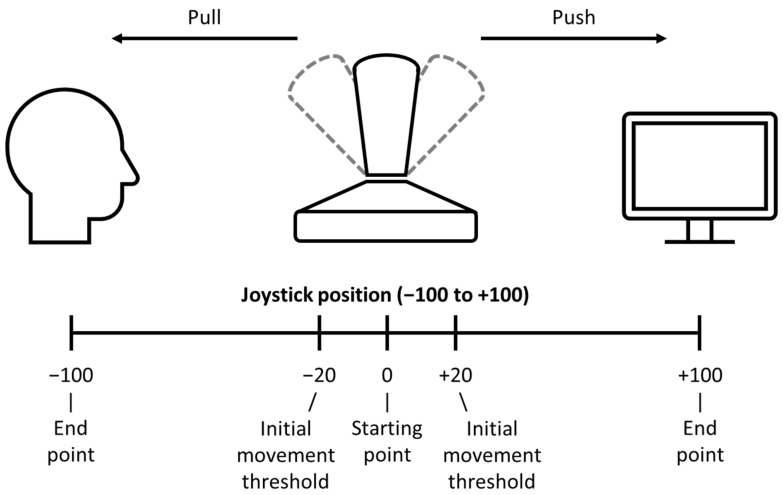
For a trial to begin, the joystick needed to be within ±2% of the starting point. Participants were instructed to slightly pivot the joystick so that it was positioned within the starting range if the trial does not begin. Once the task display appeared, participants had to either push the joystick away from them (i.e., towards the screen) or pull the joystick towards them (i.e., away from the screen). The joystick was deemed to have been moved when it had passed the initial movement threshold (20% of the entire range in each direction). Start RT is the time between the task display onset and the initial joystick movement. End RT is the time between the task display onset and the completion of the movement. Responses were registered when participants completed the movement, that is, when the joystick reached either one of the two end points.

Participants completed four runs of 60 trials. Each trial consisted of a fixation display, a task display, a blank display, a feedback display and a blank inter-trial-interval (ITI) (Figure 1). The initial fixation display remained on the screen for 500 ms and until the joystick was within the starting range (±2% of the starting point; see Figure 2).

The task display was then presented for 1500 ms or until a response was registered, and it contained a target circle (6.3° in diameter) on the left or right of a central fixation cross (11° centre-to-centre). The target circle appeared equally often in one of three equiluminant colours (orange, green and blue). One of the colours was followed by a reward of 25 cents in 80% of trials, another one of the colours was followed by a shock in 80% of trials, and the remaining colour was never followed by an outcome (neutral). The target circle had either a horizontal or vertical white line segment in it (3.8° in length). Participants were instructed to either push or pull the joystick as soon as possible, depending on the orientation of the line segment. As they pushed the joystick away from their body, the size of the target circle decreased gradually, consistent with receding motion (minimum size = 1.3° in diameter). As they pulled the joystick, the size of the target circle increased gradually, consistent with looming motion (maximum size = 18.9° in diameter). The colour-outcome mapping and line segment-response direction mapping were counterbalanced. The target colour, location and line segment orientation were fully crossed within each run, and the trials were presented in a random order. Therefore, participants were required to push and pull the joystick equally often for each target colour.

After a blank display which lasted for 1000 ms, the feedback display was presented for 1500 ms. It contained the word “Correct” or “Incorrect” depending on participants’ performance. The shock was delivered concurrently with the performance feedback on 80% of the shock trials. On 80% of reward trials, the total reward amount and the amount earned on the trial were presented together with the performance feedback. The feedback display was followed by a blank ITI lasting for 400–600 ms.

##### Test Phase

Participants completed four runs of 84 trials. Each trial consisted of a fixation display, a task display, a blank display, a feedback display and a blank ITI (Figure 1). The initial fixation display was identical to that of the training phase.

The task display was presented for 1500 ms or until a response was registered. It contained a target square (6.3° in width) and a distractor circle (6.3° in diameter), each of which appeared on the left and right of a central fixation cross (11° centre-to-centre) equally often. The target square always appeared in grey, while the distractor circle appeared in one of the three colours equally often. The distractor circle had a line segment (3.8° in length) slanted 45° to the left or right (as in, e.g., [2,25,26,27,28]). The target square had either a horizontal or vertical white line segment of equal length. The 45° line segment inside of the distractor prevented the target from being distinguishable by the presence of an internal line segment, balancing the display. Participants were instructed to either push or pull the joystick as soon as possible, depending on the orientation of the line segment in the target square. They were also explicitly told that the colour of the shapes was task-irrelevant and that they would no longer receive any rewards or shocks. Unlike in the training phase, the shapes did not change in size with joystick action. The line segment-response direction mapping was counterbalanced. The distractor colour, location and target line segment orientation were fully crossed within each run, and trials were presented in a random order.

The blank display was presented for 1000 ms, and the feedback display was presented only when participants timed out (“Too slow”) or made an incorrect response (“Incorrect”). Each trial ended with a blank ITI presented for 400–600 ms.

### 2.2. Data Analysis

Two types of response times (RTs) were recorded in the experiment. Start RT measures the time between the task display onset and the initial joystick movement. The initial joystick movement was deemed to have occurred when it had moved more than 20% of the entire range in either direction [51]. End RT was defined as the time between the task display onset and the response completion. A response was considered complete when the joystick reached either end point. Trials with start RTs faster than 200 ms were discarded from the analysis. Then, for start and end RTs separately, we excluded trials outside of 3 SDs of the conditional mean for a given participant. In keeping with Suh and Abrams [51], we were primarily interested in RT as the dependent measure, which is frequently the more sensitive indicator of biased information processing in speeded tasks of this sort (see, e.g., [2,3,26,27,28]), although we also report accuracy for completeness and because some studies demonstrate biases in both measures (see, e.g., [4,5,6,8]). We report Greenhouse-Geisser corrected *p*-values when appropriate. In the event of a non-significant interaction between the target/distractor type and the response direction, we sought to quantify the weight of evidence in favour of the absence of an interaction effect using the repeated-measures Bayesian ANOVA functionality in JASP 0.16.4.0. Specifically, we compared a null model that includes the two main effects to a model that includes both main effects plus the interaction term in order to quantify the evidence in favour of the inclusion of the interaction term; a Bayes Factor in favour of the null hypothesis (BF_01_) greater than three was taken as evidence supporting the absence of an interaction effect.

### 2.3. Results

#### 2.3.1. Training Phase

Start RT, end RT and accuracy were each subjected to a 2 × 3 repeated-measures analysis of variance (ANOVA) with the response direction (pull/approach, push/withdraw) and target type (reward, shock, neutral) as factors (Table 1). Across all three dependent measures, no significant interaction effect between the two factors was found, *F*s < 1.04, *p*s > 0.35. The absence of the interaction effect was corroborated by a repeated-measures Bayesian ANOVA. The BF for the inclusion of the interaction term was BF_01_ = 4.68 for start RT, BF_01_ = 9.32 for end RT and BF_01_ = 8.55 for accuracy.

There was no significant main effect of the response direction on start RT, *F*(1, 37) = 0.51, *p* = 0.48, suggesting there was no difference between the latencies in initiating a pull/approach and a push/withdraw response. However, participants were faster to complete the pull/approach response (*M* = 743 ms, *SD* = 123 ms) than the push/withdraw response (*M* = 774 ms, *SD* = 152 ms), as indicated by a significant main effect of the response direction on end RT, *F*(1, 37) = 9.82, *p* = 0.003, η²_p_ = 0.21. There was no significant main effect of target type on either start or end RTs, *F*s < 2.15, *p*s > 0.14.

There was a significant main effect of target type on accuracy, *F*(2, 74) = 4.29, *p* = 0.026, η²_p_ = 0.1, suggesting that participants learned the target-outcome associations. Accuracy was higher in reward target trials (*M* = 98.6%, *SD* = 0.02%) than in shock target trials (*M* = 97.3%, *SD* = 3.2%), *t*(37) = 2.64, *p* = 0.012, *d* = 0.44. These accuracies were not significantly different from accuracy on neutral trials (*M* = 98.3%, *SD* = 1.4%), *t*s < 1.83, *p*s > 0.07. There was no significant main effect of the response direction, *F*(1, 37) = 3.05, *p* = 0.09.

#### 2.3.2. Test Phase

Start RT, end RT, and accuracy were subjected to a 2 × 3 repeated-measures ANOVA with the response direction (pull/approach, push/withdraw) and distractor type (reward, shock, neutral) as factors (Table 2). Across all three dependent measures, there was no significant interaction effect between the two factors, *F*s < 1.49, *p*s > 0.23, suggesting that distractor valence does not modulate the response direction. A repeated-measures Bayesian ANOVA revealed that the BF for the inclusion of the interaction term was BF_01_ = 4.21 for start RT, BF_01_ = 2.89 for end RT and BF_01_ = 5.32 for accuracy.

There was no significant main effect of the response direction on start RT, *F*(1, 37) = 1.07, *p* = 0.31, but a significant effect on end RT, *F*(1, 37) = 9.82, *p* = 0.003, η²_p_ = 0.21. Again, latencies to initiate a pull/approach and a push/withdraw response were comparable, but participants were faster to complete the pull/approach response than the push/withdraw response.

There was a significant effect of the distractor type on start RT, *F*(2, 74) = 3.65, *p* = 0.031, η²_p_ = 0.09, but not on end RT, *F*(2, 74) = 2.78, *p* = 0.07. Although there were differences in the latencies in initiating a response, participants took comparable amounts of time to complete their responses. They were faster to initiate a response when there was a reward distractor (*M* = 633 ms, *SD* = 94 ms) than when there was a shock distractor (*M* = 640 ms, *SD* = 96 ms), *t*(37) = 2.4, *p* = 0.022, *d* = 0.41. These start RTs were not significantly different from the RT on the neutral trials (*M* = 639 ms, *SD* = 91 ms), *t*s < 1.84 *p*s > 0.07. No significant effects were found on accuracy, *F*s < 3.56, *p*s > 0.06.

### 2.4. Discussion

Experiment 1 demonstrates that, despite evidence of valence learning, particularly with respect to reward-associated stimuli, there was no indication of a response direction modulation by the reward- and threat-signalling distractors. In the test phase, participants were faster on reward than on shock distractor trials, regardless of whether they were required to push or pull the joystick to withdraw from or approach the target. Although prior reward associations may have invigorated the responses more generally, we see no evidence of either stimulus-outcome association biasing responses in a particular direction.

One explanation for the absence of a critical interaction effect may be that the way we manipulated the approach-avoidance action was not sufficiently sensitive. It is possible that participants did not perceive the target size change as the target approaching/getting closer to them or withdrawing/receding from them. In addition, the manner in which approach and avoidance actions were defined may have been ambiguous. Classical experimental definitions of approach and avoidance behaviour have tended to focus on the idea of drawing a stimulus closer vs. pushing it away (e.g., [20,49]). However, arm flexion can be conceived of as a withdrawal response (e.g., removing the hand from a hot surface) whereas arm extension can be conceived of as an approach response (e.g., reaching for dollar bills on the ground) [52,53,54,55]. This ambiguity in the manner in which pull and push motions could be mapped to approach and avoidance responses might have reduced the strength of learning in our Experiment 1 [56,57]. In addition, a manikin task, which we employed in Experiment 2, is generally considered a more sensitive measure of approach-avoidance tendencies [54,58].

It could also be asked how robustly participants learned the colour-outcome relationships in Experiment 1. The only evidence for learning was a slightly more accurate response to reward-associated targets during training and a facilitatory effect of previously reward-associated distractors during the test phase, the latter of which ran counter to what would have been expected from elevated distractor interference. Experiment 2 provided an opportunity to probe for differential response biases for previously reward- and threat-associated stimuli in the context of more robust evidence for the learning of the colour-outcome contingencies.

## 3. Experiment 2

Experiment 2 continues to examine the modulatory effect of reward and threat histories on the response direction. We made two major changes to the task design from Experiment 1: (1) we fully crossed the physical action (i.e., arm flexion/pulling and extension/pushing) and the response direction (i.e., approach and avoidance) and (2) we used a manikin to improve sensitivity.

### 3.1. Methods

#### 3.1.1. Participants

A different sample of 31 participants (18 females; mean age = 20.4 years) were recruited from the Texas A&M University community. All participants satisfied the criteria mentioned in Experiment 1.

#### 3.1.2. Apparatus

Identical to Experiment 1, with the exception that responses during the training phase were entered using a MilliKey response box.

#### 3.1.3. Procedure

##### Shock Calibration

Identical to Experiment 1.

##### Training Phase

**Figure 3:**
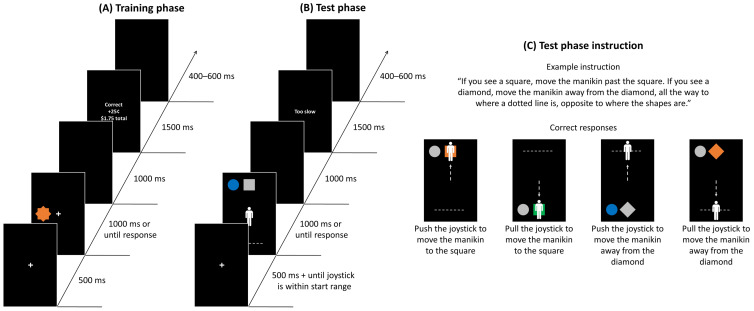
Sequence of trial events for the (**A**) training and (**B**) test phases in Experiment 2. (**C**) Test phase example instruction and correct responses that correspond to the instruction. The vertical dotted line with an arrow depicts the movement of the manikin resulting from the correct joystick action and is for illustration purposes (did not actually appear in the experiment).

Identical to Experiment 1, with the following exceptions (Figure 3A). Participants completed three runs of 60 trials. The task display contained a star-like shape target either on the left or right side, 6.9° centre-to-centre from a central fixation cross. The target was produced by overlaying a square (5.1° in width) on top of a diamond of equal size. It appeared equally often in one of three equiluminant colours (orange, green and blue). One of the colours was followed by a reward of 25 cents on every trial, another one of the colours was followed by a shock on every trial, and the remaining colour was never followed by an outcome (neutral). Participants were instructed to press the left key on the response box if the target appears on the left side, and the right key if it appears on the right side, with their right index and middle finger, respectively.

##### Test Phase

Participants completed four runs of 96 trials. Each trial consisted of a fixation display, a task display, a blank display, a feedback display and a blank ITI (Figure 3B). The initial fixation display remained on the screen for 500 ms and until the joystick was within the starting range (±2% of the starting point; see Figure 2). The task display was presented for 1500 ms or until a response was registered. It contained a manikin (2.1° × 5.3°) at the centre of the screen, a target shape, and a distractor circle (5.1° in diameter) to the left or right of the target shape (6.8° centre-to-centre) and a dotted line (13.8° in length). The target could be either a square (5.1° in width) or a diamond of equal size. The target and distractor shapes and dotted line could appear either in the top half or bottom half of the screen (16.2° from the centre of the screen) equally often, and they never appeared in the same half. The target/distractor shape appeared in one of four colours (orange, green, blue and grey). If the target was coloured, then the distractor was rendered in grey and vice versa.

Participants were instructed to either push or pull the joystick to move the manikin as soon as possible, depending on the target shape. When they pulled, the manikin moved toward the bottom of the screen. When they pushed, the manikin moved toward the top of the screen. For example, if a participant was instructed to move the manikin toward the diamond target and away from the square target, and if the square target appeared in the top half of the screen and the dotted line appeared in the bottom half, the participant had to pull the joystick to move the manikin toward the bottom of the screen, all the way to down to where the dotted line was located (see Figure 3C for other examples of instructions). Participants were also explicitly told that shape colours are task-irrelevant, and they will no longer receive either reward or shock. The target shape-response direction mapping was counterbalanced. The target/distractor colour, target/distractor, and dotted line locations were fully counterbalanced within each run, and the trials were presented in a random order.

The blank display was then presented for 1000 ms. The feedback display was presented only when participants failed to make a response in time (“Too slow”) or made an incorrect response (“Incorrect”). Each trial ended with a blank ITI lasting for 400–600 ms.

### 3.2. Data Analysis

Training phase RTs faster than 200 ms or exceeding 3 SDs of the conditional mean for a given participant were excluded from the analysis. The test phase RT processing was identical to that of Experiment 1. We report Greenhouse-Geisser corrected *p*-values when appropriate.

### 3.3. Results

#### 3.3.1. Training Phase

RT and accuracy were subjected to a one-way repeated-measures ANOVA with the target types (reward, shock, neutral) as factors (Table 3). No significant effects were found for RT, *F*(2, 60) = 1.88, *p* = 0.18, but there was a marginal effect on accuracy, *F*(2, 60) = 3.73, *p* = 0.056, η²_p_ = 0.11. The accuracy was numerically lower on shock target trials (*M* = 98.9%, *SD* = 2.08%) than on reward (*M* = 99.7%, *SD* = 0.62%) and neutral (*M* = 99.7%, *SD* = 0.63%) target trials, *t*s < −1.97, *p*s > 0.054. The accuracies on reward and neutral target trials were comparable, *t*(30) = 0.01, *p* = 0.99.

#### 3.3.2. Test Phase

Start RT, end RT, and accuracy were subjected to a 2 × 3 repeated-measures ANOVA with the response direction (approach, withdraw) and target/distractor type (reward, shock, neutral) as factors, performed separately for trials on which the targets were valent and those on which the distractors were valent (Table 4). On the valent target trials, there was a significant main effect of the response direction on both start RT and end RT, *F*s > 13.85, *p*s < 0.001. Participants were faster to initiate and complete the response which required them to move the manikin towards the valent target. There was also a significant main effect of the response direction on the accuracy. It was higher when participants were required to move the manikin away from the valent target than when they had to move the manikin towards the valent target, *F*(1, 30) = 5.02, *p* = 0.033, η²_p_ = 0.14. Across all three dependent measures, there was no main effect of target type or interaction, *F*s < 0.65, *p*s > 0.11. The absence of the interaction effect was supported by a repeated-measures Bayesian ANOVA. The BF for the inclusion of the interaction term was BF_01_ = 10.3 for start RT, BF_01_ = 5.74 for end RT and BF_01_ = 5.95 for accuracy.

On valent distractor trials, there was a main effect of the response direction on both start RT and end RT, *F*s > 18.79, *p*s < 0.001. Participants were again faster to initiate and complete the response which required them to move the manikin towards the grey target. There was no significant effect on accuracy, *F*(1, 30) = 0.72, *p* = 0.4. There was a main effect of distractor type on start RT and end RT, *F*s > 6.16, *p*s < 0.01. Participants were faster to start moving the manikin when there was a neutral distractor (*M* = 620.7 ms, *SD* = 83.2 ms) than when there were reward (*M* = 632.9 ms, *SD* = 87.8 ms) and shock (*M* = 627.7 ms, *SD* = 79 ms) distractors, *t*s > 2.42, *p*s < 0.021, *d*s > 0.43. Start RTs on reward and shock distractor trials did not differ, *t*(30) = 1.22, *p* = 0.23. Likewise, participants were faster to finish moving the manikin when there was a neutral distractor (*M* = 763.4 ms, *SD* = 93.3 ms) than when there were reward (*M* = 785.5 ms, *SD* = 99.6 ms) and shock (*M* = 777.9 ms, *SD* = 94.7 ms) distractors, *t*s > 4.12, *p*s < 0.001, *d*s > 0.73. End RTs on reward and shock distractor trials did not differ, *t*(30) = 1.5, *p* = 0.15. Importantly, across all three dependent measures, there was no interaction effect between the two factors, *F*s < 2.33, *p*s > 0.1. The BF for the inclusion of the interaction term was BF_01_ = 8.63 for start RT, BF_01_ = 4.82 for end RT and BF_01_ = 1.1 for accuracy.

### 3.4. Discussion

Similar to Experiment 1, Experiment 2 also demonstrates that, despite evidence of valence learning, there was no indication of response direction modulation by reward- and threat-signalling stimuli. In the test phase, participants were generally faster to move the manikin closer to the target, irrespective of whether it was the target or the distractor that was valent. We also observed an effect of the distractor type, independent of the response direction. Participants were slower to initiate and complete their responses on reward and shock distractor trials than neutral distractor trials, consistent with an effect of distractor interference magnified by colour-outcome association. Such elevated interference by valent stimuli provides robust evidence that participants learned the colour-outcome associations, replicating value and threat-modulated attentional capture [1,2,3,4,5,6]. Even using the manikin task, which is considered more sensitive in capturing the valence-action bias [54,58], in Experiment 2, we see no evidence that reward- and threat-signalling stimuli modulate the response direction, even in a context in which there is a reliable distractor cost consistent with valence-modulated attentional capture. Regardless of whether the valent stimulus was a target or a distractor, reward- and shock-associated colours did not differently facilitate approach and avoidance responses.

## 4. General Discussion

The present pair of experiments examined whether the involuntary influence of reward and threat histories extends to response selection. Specifically, we hypothesised that task-irrelevant distractors previously associated with reward or aversive outcomes would not only capture attention but also activate approach and avoidance responses, respectively, given prior findings concerning valence-action biases and a common neural profile between attention and motor control. Across the two experiments, our results revealed a general valence effect without an interaction effect; despite the effective valence learning and the evidence of attentional biases in favour of valent distractors, reward- and threat-signalling distractors did not modulate the response direction. Although the evidence that participants learned the colour-outcome relationships was more tenuous in Experiment 1, in Experiment 2 we replicate the evidence for value- and threat-modulated attentional capture, which can only be explained as resulting from colour-based associative learning. However, at no point were responses selectively advantaged when an approach response was required in the presence of a previously reward-associated stimulus or an avoidance response in the presence of an aversively conditioned stimulus.

This lack of interaction between the valence and response bias was also evident when these stimuli were currently predictive of their associated outcome during training. However, it is unclear at what point participants acquired the stimulus-outcome relationships during training. As in prior studies of value- and threat-driven attentional capture, our experimental design was primarily focused on identifying learning-dependent effects in the test phase (e.g., [2,3,5,6,8,19,25,26,27,28]), which is where we see our most robust evidence for the learning of the colour-outcome pairings (especially in Experiment 2), and so we restrict our conclusions specifically to the test phase and the biases involuntarily evoked by task-irrelevant stimuli.

Our observed results suggest that reward and threat histories have comparable effects on attention and response selection; stimuli previously associated with valent outcomes invigorate attentional orienting and execution of response, but their effects do not extend to response selection. Using a manikin task, Hoofs, et al. [59] have demonstrated that stimuli indicative of reward and aversive outcomes enhance task performance, independent of the response direction. In their study, aversive outcomes could be avoided by making a correct response. Such manipulation of aversive outcomes may not be considered truly aversive but rather rewarding via negative reinforcement [60]. In the current study, even without an opportunity to avoid an aversive outcome, we show that the valence and the response direction have independent effects. We do not see evidence for an interaction between the valence and the response direction, regardless of whether the valent stimulus was a target or a distractor, extending the findings of Hoofs, Carsten, Boehler and Krebs [59].

Suh and Abrams [51] reported that the response direction modulates value-driven attention. In contrast to the present finding that reward- and threat-signalling stimuli disrupt attention and action regardless of the response direction, they demonstrated that valence-action-compatible distractors bias attention, such that stimuli that come to predict high-value via an approach response and those that come to predict low-value via an avoidance response during training bias attention. It is intriguing to have obtained a different pattern of results with respect to the relationship between the response direction and attention, provided that the training task employed in their study is very similar to the one we used in Experiment 1. One possibility is that the nature of stimulus-response bindings with respect to approach and avoidance actions influences how well stimulus-outcome relationships are learned [51], while our findings suggest that, once a stimulus-outcome relationship is learned, the resulting attentional bias does not imply a corresponding bias with respect to the behaviour evoked by the stimulus. Given that their training task involved twice the number of trials, it is also possible that a selection history induced valence-action bias requires more extensive training. Further investigation is warranted to identify the factors that generate this diverging pattern of results.

The absence of an interaction between the valence and the response direction suggests that automatic modulation of the response direction by the valence is not as reflexive as originally suggested [61,62]. Indeed, the expression of automatic approach and avoidance actions in response to appetitive and aversive stimuli has been shown to depend on a conscious evaluation of the target valence and task-relevance of the feature participants are responding to [54,63]. However, in the present study, participants only implicitly appraised the colour-outcome associations, and colour, which signalled reward and aversive outcomes, was never a task-relevant feature.

The valence of the distractors did not interact with the direction of response in the present study, while prior studies have demonstrated an influence of the reward history of distractors on inhibitory control [17,19]. One possible explanation is that inhibitory control and response selection rely on different underlying mechanisms with respect to their relationship with attentional biases. Inhibitory control and response selection are typically assessed using the go/no-go or stop signal task and the approach-avoidance task, respectively. Dietary training programmes that are based on these paradigms have demonstrated that go/no-go tasks are more effective in promoting healthier eating behaviour than approach-avoidance tasks [64,65], indicative of a potential distinction between the two processes. While inhibitory control concerns the execution or withholding of an action, approach-avoidance always requires the execution of an action. It is possible that such a global invigoration of action discounts directionality.

It is worth noting that, in the present study, reward was manipulated as a secondary reinforcer (money), whereas aversive outcomes were manipulated as a primary punisher (electric shock). When directly compared, the stimuli associated with these outcomes have comparable effects on attentional bias as measured both via behaviour and with respect to neural correlates [8]. Comparable behavioural effects were replicated using both primary rewards and punishers equated for valence [66]. Furthermore, to the degree that stimuli associated with primary and secondary reinforcers/punishers are processed differently in the brain, we might have expected such outcome-related differences to facilitate differential response biases, which we did not find evidence for. It is therefore unlikely that our results were influence by the choice of reward and aversive outcome used in conditioning.

## 5. Conclusions

In conclusion, the present study demonstrates that, while reward and threat histories shape learning-dependent attentional biases, their influence does not extend to response selection. Stimuli predictive of reward and threat stimulated action, but they did not modulate its direction such that reward-signalling stimuli preferentially activate an approach response while threat-signalling stimuli preferentially activate an avoidance response. This challenges the idea that the link between the valence and the response tendencies is inherent in nature and also suggests that involuntary biases in attention and behaviour are subserved by independent underlying mechanisms.

## Figures and Tables

**Table 1:** Mean start RT, end RT and accuracy as a function of target type and response direction in the training phase of Experiment 1. Numbers in the parentheses represent the standard deviations.

	Start RT (ms)			End RT (ms)			Accuracy (%)		
	Reward	Shock	Neutral	Reward	Shock	Neutral	Reward	Shock	Neutral
Pull/ approach	601.9 (92)	609.4 (98.1)	611.8 (94.1)	736 (120.6)	744.7 (127.5)	746.8 (122.8)	99 (1.7)	97.7 (3.9)	98.4 (2.4)
Push/ withdraw	602.8 (98)	604.6 (111.5)	601.9 (99.3)	766.8 (140.5)	780.4 (165.9)	775.8 (153.1)	98.2 (2.9)	97 (3.5)	98.2 (2.3)

**Table 2:** Mean start RT, end RT and accuracy as a function of distractor type and response direction in the test phase of Experiment 1. Numbers in the parentheses represent the standard deviations.

	Start RT (ms)			End RT (ms)			Accuracy (%)		
	Reward	Shock	Neutral	Reward	Shock	Neutral	Reward	Shock	Neutral
Pull/ approach	644.9 (85.4)	642.7 (96)	649.5 (96.8)	753 (108)	751.5 (119.6)	757.3 (119.7)	98.5 (2.5)	99 (2.8)	98.3 (2.3)
Push/ withdraw	642.7 (103.3)	632.3 (100.1)	641.5 (103.5)	785.5 (138.7)	774.1 (134.5)	781.9 (139.6)	97.7 (3)	98.5 (1.9)	98.3 (2.1)

**Table 3:** Mean RT and accuracy for each target type in the training phase of Experiment 2. Numbers in parentheses represent the standard deviations.

	Reward	Shock	Neutral
RT (ms)	318.3 (42.4)	322.9 (54)	316.9 (43.7)
Accuracy (%)	99.7 (0.6)	98.9 (2.1)	99.7 (0.6)

**Table 4:** Mean start RT, end RT and accuracy for each target type (top) and distractor type (bottom) in the test phase of Experiment 2. Numbers in the parentheses represent the standard deviations.

**Valent Target**									
	**Start RT (ms)**			**End RT (ms)**			**Accuracy (%)**		
	**Reward**	**Shock**	**Neutral**	**Reward**	**Shock**	**Neutral**	**Reward**	**Shock**	**Neutral**
Approach	607.1 (79.7)	611.7 (77.4)	610.8 (84.5)	758.1 (101.4)	753.5 (90)	753.8 (94.3)	98 (3.5)	98.1 (3)	98.7 (2.6)
Withdraw	638.4 (97.1)	642 (88.9)	641.7 (95.8)	780.3 (107.8)	784.7 (95.4)	785.8 (106.9)	99.1 (2.2)	98.5 (2.9)	99.2 (2.3)
**Valent Distractor**									
	**Start RT (ms)**			**End RT (ms)**			**Accuracy (%)**		
	**Reward**	**Shock**	**Neutral**	**Reward**	**Shock**	**Neutral**	**Reward**	**Shock**	**Neutral**
Approach	619.8 (83.4)	611.7 (80)	607.9 (79.9)	769.6 (97.3)	758.6 (95.8)	751.4 (91.5)	98.3 (3)	98.4 (3.5)	99.1 (2.4)
Withdraw	649.8 (97.7)	645.4 (86.3)	636.7 (92.3)	800 (111.1)	797.5 (102.3)	776.6 (102.2)	99.1 (2.3)	99 (2.2)	98.6 (2.3)

## Data Availability

The data are available upon a request made to the lead author, Haena Kim (kimhannah@uchicago.edu), and will be provided under the provision that the data be used strictly for academic research purposes and not be shared with others without the express written approval of the lead author.
